# Supplementary material for: Association between oral microbiome and seven types of cancers in East Asian population: a two-sample Mendelian randomization analysis
Source: Front Mol Biosci. 2023 Nov 21;10:1327893. doi: 10.3389/fmolb.2023.1327893 (PMC10702768; doi:10.3389/fmolb.2023.1327893)
Supplement: Supplementary file 1 [file DataSheet1.ZIP › Supplementary Materials/MR plots of saliva/saliva═╝/Pancreatic cancer/pheno.333_to_pancreatic cancer_forest.pdf]

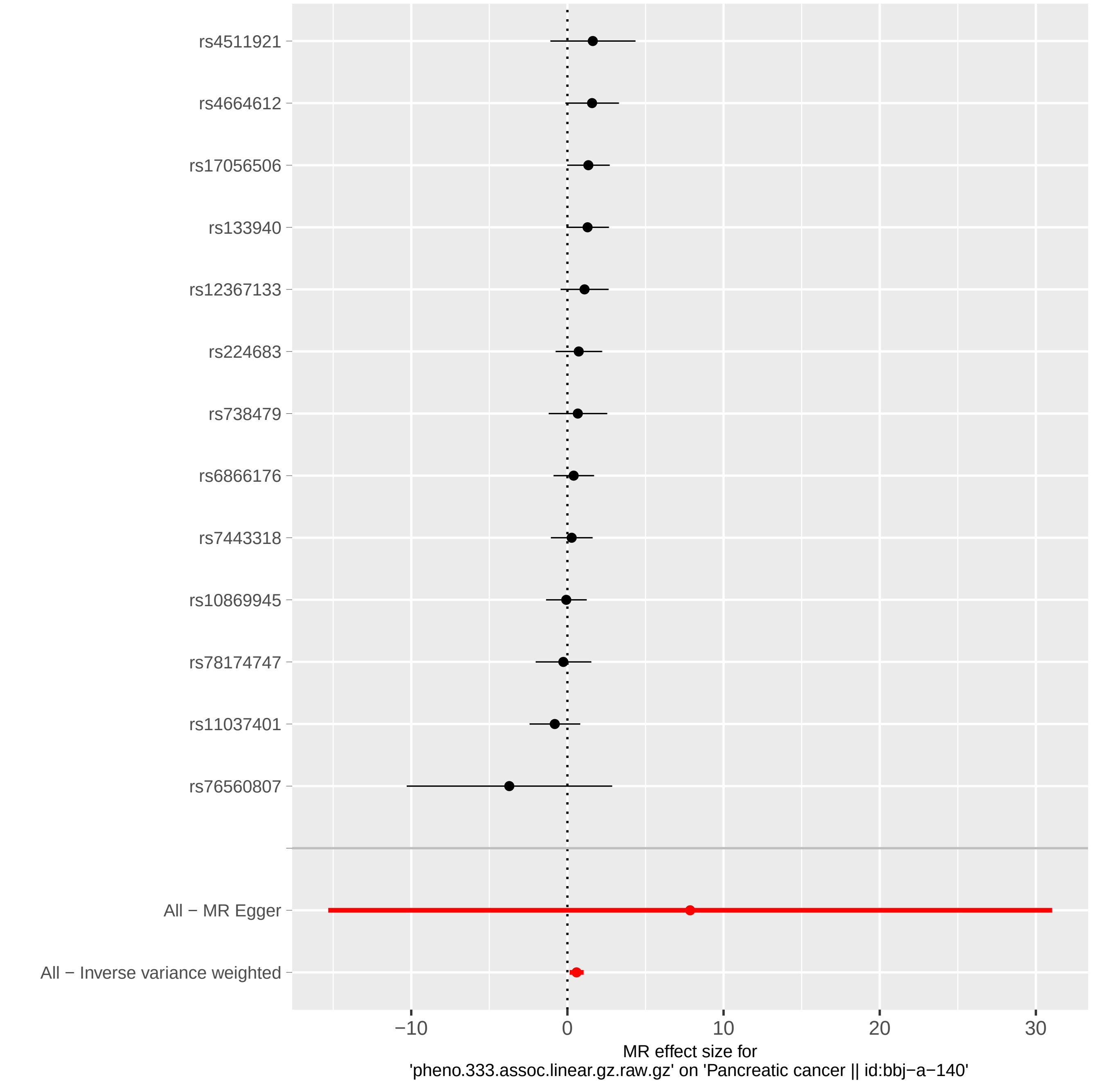
rs4511921
rs4664612
rs17056506
rs133940
rs12367133
rs224683
rs738479
rs6866176
rs7443318
rs10869945
rs78174747
rs11037401
rs76560807
All − MR Egger
All − Inverse variance weighted
−10
0
10
20
30
MR effect size for
'pheno.333.assoc.linear.gz.raw.gz' on 'Pancreatic cancer || id:bbj−a−140'
